# Convergence of Organ‐on‐a‐Chip and Freeform Printing of Sacrificial Poly(2‐cyclopropyl‐2‐oxazoline) Enables the Generation of Perfusable Endothelialized Channels in Hydrogels

**DOI:** 10.1002/marc.202500802

**Published:** 2026-01-21

**Authors:** Giulia Maria Di Gravina, Patrick Kuntschke, Zeno Guttenberg, Philipp Linke, Katja P. Schumann, Meike N. Leiske, Thomas Scheibel, Matthias Ryma

**Affiliations:** ^1^ Department of Biomaterials University of Bayreuth Bayreuth Germany; ^2^ Chair for Functional Materials in Medicine and Dentistry at the Institute for Functional Materials and Biofabrication University of Würzburg and Bavarian Polymer Institute Würzburg Germany; ^3^ Ibidi GmbH Gräfelfing Germany; ^4^ Macromolecular Chemistry University of Bayreuth Bayreuth Germany; ^5^ Bavarian Polymer Institute (BPI) University of Bayreuth Bayreuth Germany; ^6^ Bayreuth Center for Colloids and Interfaces (BZKG) University of Bayreuth Bayreuth Germany; ^7^ Bayreuth Center for Molecular Biosciences (BZMB) University of Bayreuth Bayreuth Germany; ^8^ Bayreuth Center for Material Science (BayMAT) University of Bayreuth Bayreuth Germany; ^9^ Faculty of Medicine University of Würzburg Würzburg Germany

**Keywords:** endothelialization, freeform printing, microvascular networks, organ‐on‐a‐chip, thermoresponsive polymers

## Abstract

The fabrication of physiologically relevant microvascular networks remains a major challenge in Organ‐on‐a‐chip (OoC) technologies, largely due the dependence on plastic materials and the generation of channels with non‐physiological, rectangular cross‐sections. Here, we present a novel approach for the in‐situ generation of perfusable micro‐vessels within OoC platforms using freeform printing (FFP) of the thermo‐responsive poly(2‐cyclopropyl‐2‐oxazoline) (PcycloPrOx). We integrated FFP with a fluidic custom‐designed OoC device to directly print suspended sacrificial vascular templates, enabling the creation of circular cross‐section channels with resolutions down to 200 µm, without post‐processing. Following hydrogel casting and template dissolution, green fluorescent protein human umbilical vein endothelial cells (GFP‐HUVECs) were seeded into the channels and cultured under continuous perfusion (unidirectional and bidirectional) for 7 days. Confocal fluorescence microscopy revealed rapid endothelialization, with a confluent monolayer established by day 3. By day seven, immunostaining confirmed expression of endothelial markers CD31 and VE‐cadherin, indicating proper endothelialization. This work demonstrates the first functional application of PcycloPrOx‐based FFP for OoC vascularization and provides a scalable, automatable strategy for engineering perfusable, endothelialized microvascular 3D tissue models. Our platform offers new opportunities for vascularized organ‐on‐chip models in drug screening and disease modelling.

## Graphical abstract

We employ freeform printing of thermo‐responsive poly(2‐oxazoline) to directly fabricate suspended sacrificial vascular templates within Organ‐on‐a‐chip devices. This enables circular, perfusable channels down to 200 µm without post‐processing. Subsequent endothelialization with GFP‐HUVECs under perfusion yields confluent, marker‐expressing microvessels, providing a scalable strategy for vascularized OoC platforms for drug screening and disease modelling.

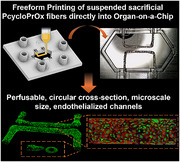

## 1 Introduction

Organ‐on‐a‐Chip (OoC) technologies have recently emerged as a groundbreaking approach in biomedical research, aiming to recapitulate the cellular microenvironment in a controllable way, by improving the predictability for in vivo tissue/organ behavior [1]. In particular, OoC models enable to define and control the geometry and matrix material in which the cells are implemented, offering a more accurate and efficient alternative to traditional in vitro and in vivo studies and, consequently, a great potential for revolutionizing the drug development pipeline and the comprehension of disease mechanism [2].

As an emerging technology, OoC systems are already commercially available in a variety of forms, including microfluidic platforms with vascular channels, multi‐organ chips for simulating systemic interactions, and modular systems for customizable applications. However, these systems face significant technical drawbacks, such as the use of artificial components like plastic and membranes, which can limit physiological tissue mimicry. For example, polydimethylsiloxane (PDMS) is often used as microfluidic device base material [3], which is far too stiff to faithfully mimic the mechanical properties of in vivo extracellular matrix (ECM). Further, the morphological, chemical, and biological properties of ECM are not well represented by PDMS. Similarly, the use of artificial membranes to separate different compartments (e.g., lung side from vessel side) limits the direct cell–cell interactions and reduces the fidelity of physiological crosstalk between tissues [4]. Moreover, microfluidic systems are built through conventional fabrication methods such as photolithography, through which the channels have rectangular cross sections and flat walls. Hence, the geometry of tubular‐structured tissue with circular cross sections, such as blood vessels, is not represented well, affecting the fluid flow behavior and consequently imposing a not‐physiological shear stress stimulus to the cells [5].

To face these problems, recently new techniques have been explored in the field of OoC to fully integrate embedded and perfusable channels with a micro‐scale circular cross‐section into soft materials, such as hydrogels, able to closely mimic the ECM properties. Methods based on sacrificial templating coupled with extrusion‐based 3D printing represents a promising complementary technique [6]. Particularly, extrusion‐based 3D printing of sacrificial materials called as Freeform Printing (FFP) has shown to allow the manufacturing of complex geometries and branched features inside hydrogels, in which hollow structures like channels easily collapse [7]. FFP can be categorized in two groups. The first one, more common and mostly known, is based on a supporting matrix with specific rheological properties that enables the deposition of a sacrificial material preventing its collapse. The supporting matrix is then crosslinked, and the sacrificial template can be dissolved through different stimuli (e.g., temperature changes), leaving behind perfusable channels within the solidified hydrogel [8].

In the second group, the sacrificial material is self‐supporting, which means that it must rapidly solidify upon extrusion at ambient conditions – for this a glass transition temperature above room temperature is needed – and possess good mechanical properties once printed to keep the structural integrity [9, 10]. During the printing, these two properties allow the deposition of freestanding micro‐scale filaments forming complex 3D networks, on which a hydrogel precursor solution is cast and then crosslinked. Finally, the sacrificial filaments are washed‐out creating in this way the channels inside the hydrogels.

This second category is more uncommon, probably due to a narrow plethora of materials. Indeed, applied to biological purposes, this technique has been shown by using two different materials. The first one is a carbohydrate glass, composed by a mixture of sucrose, glucose, and dextran, which has been applied to the fabrication of vascular channels [11] and renal proximal tubules [12]. However, to improve stability during hydrogel gelation and prevent premature dissolution, the printed filaments were dip‐coated with a degradable polyester (poly lactide‐co‐glycolyde) (PLGA).

The second material is poly(2‐cyclopropyl‐2‐oxazoline) (PcycloPrOx), a thermo‐responsive polymer that is water‐soluble below 25°C and exhibits intrinsic biocompatibility as well as stealth behavior, eliminating the need for additional surface coatings. In the work of Mair et al. [13], freestanding microscale filaments of PcycloPrOx forming complex 3D networks were successfully deposited, and a preliminary biological validation was demonstrated. However, no specific application of this technique was proposed at that stage.

In this work, we present for the first time an approach based on the FFP of PcycloPrOx to directly print suspended networks of circular cross‐section, microscale channels within a perfusable hydrogel‐based OoC. This method addresses two major limitations of current OoC systems – namely, the reliance on plastic materials such as PDMS and the generation of rectangular cross‐section channels – while offering additional advantages. These include greater design flexibility for vascular patterns and a rapid, straightforward fabrication process enabled by the direct printing of PcycloPrOx filaments, facilitating the scalable production of vascularized and perfusable OoC platforms.

## 2 Results

### 2.1 Freeform Printing of PcycloPrOx Filaments

**FIGURE 1:**
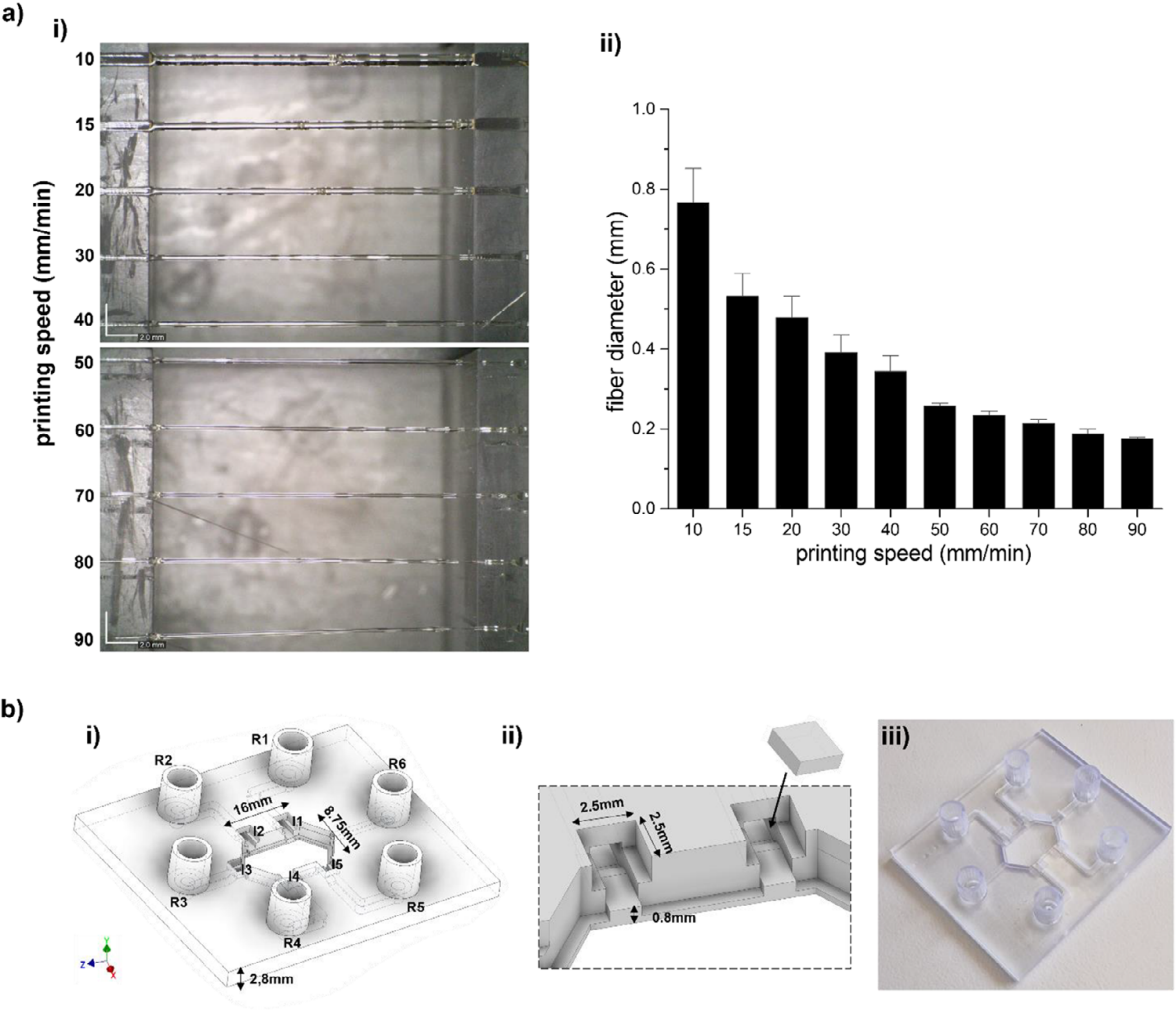
a) printed PcycloPrOx filaments by varying the printing speed (i) and quantification of the fiber diameters (ii) (n = 9). b) Perfusion OoC: i) design of the chip: central chamber, with six reservoirs (R1–R6) and five inlets (I1–I5); ii) zoom at the inlet in which is shown both the area conceived for the deposition of PcycloPrOx fibers and the 3D‐printed insert placed in the following area to close it once the printing process is terminated; iii) perfusion OoC fabricated via DLP printer.

As described in a previous study [13], FFP of PcycloPrOx allows the printing of freestanding fibers with complex geometries thanks to its quick solidification after exiting the nozzle. In this work, based on the use of a pressure‐based in‐house made printer, three different printing parameters – i.e., pressure, printing velocity, and temperature – were systematically investigated to understand their influence on the filament diameter size. As a result, the printing speed was the main parameter affecting the diameter tuning. Therefore, we repeated the analysis of the filament diameter with a piston‐based printer by varying only the printing speed between 10 and 90 mm/min and by keeping the temperature and piston extrusion – equivalent to the printing pressure – constant. As shown in Figure 1a‐i, linear PcycloPrOx filaments were printed between two walls with a distance equal to 20 mm showing the possibility to print in‐air fibers with a considerable length without the need for additional supports. The size of the fiber diameter ranged from 0.8 to 0.2 mm by decreasing the printing speed (Figure 1a‐ii), allowing to get micro‐channels in a wide processing window.

### 2.2 Development of a Micro‐Fluidic Chip Suitable for Freeform Printing

A specialized fluidic organ‐on‐chip system was developed with the following features: i) allowing the direct printing of suspended sacrificial filaments inside the chamber; ii) the perfusion of the formed channel networks for cell culture; iii) enabling microscopy studies.

The design consists in a central hexagonal chamber (16 mm x 875 mm x 28 mm) connected with five channels and relative inlets and reservoirs (Figure 1b‐i). Each inlet (I1‐I5) presents an area (Figure 1b‐ii) where the nozzle can deposit and attach the initial and final part of the filament during the printing process. This allows to place and fix the PcycloPrOx structures directly inside the chamber. The deposition of the filament from one inlet to the other is performed according to the Gcode, without touching the bottom part of the chip thanks to the fast solidification of PcycloPrOx. Once the printing process is terminated, the area is sealed with an 3D‐printed insert with the same shape, closing in this way the channel.

Regarding the perfusion, the shape of the reservoirs was designed to allow either the attachment of perfusion tubing in case of unidirectional perfusion by using of a pump or as media container in case of bidirectional perfusion via rocking on a tilting device.

To enable the visualization of the chamber and its microscopy during and after endothelialization, an observation window at the bottom and top of the chamber was conceived. Finally, a sixth reservoir was added in the design as inlet for injection of pre‐solution hydrogel and media.

### 2.3 Channel Generation inside OoC With Freedom of the Design

**FIGURE 2:**
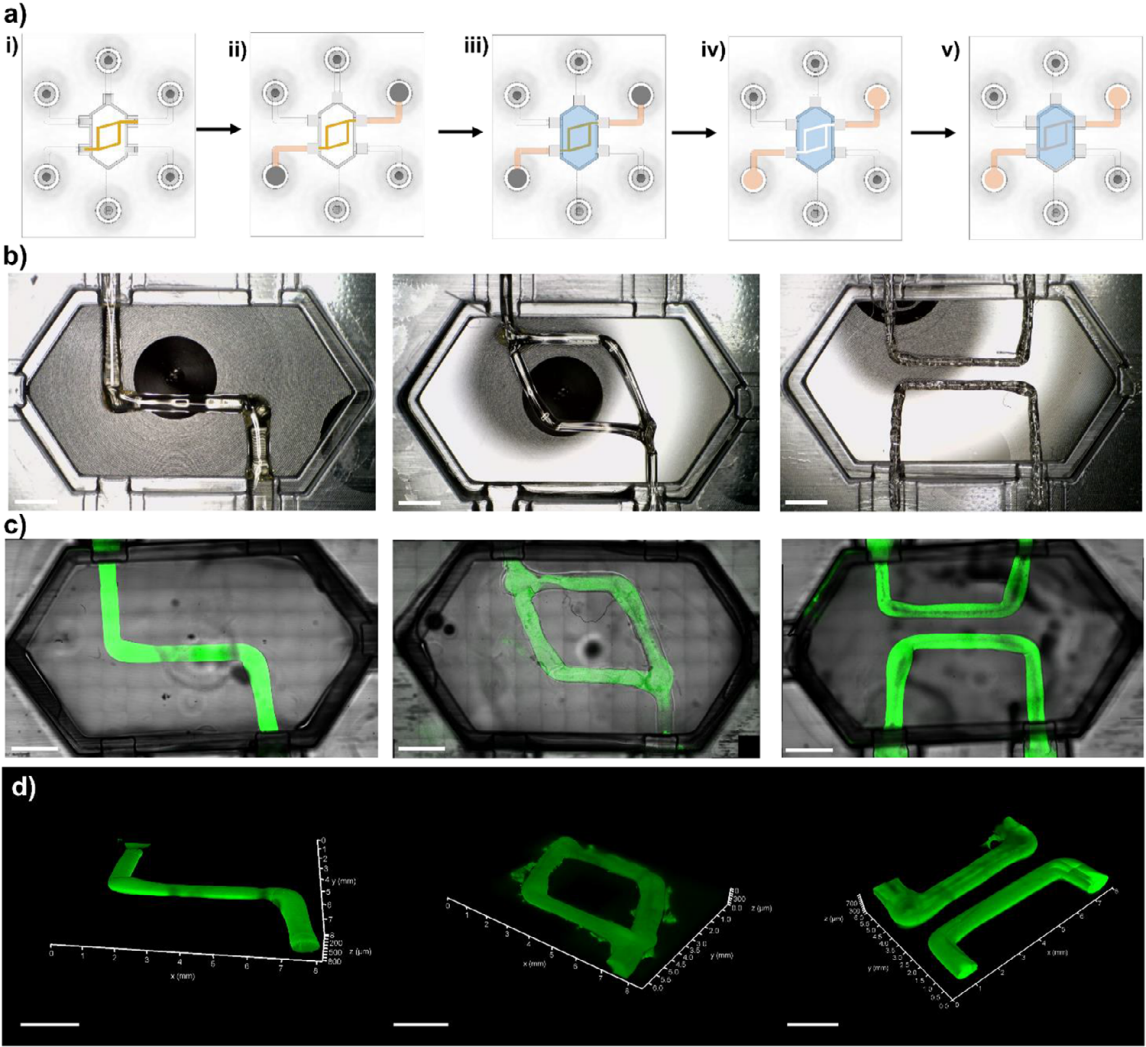
Channel generation inside the chips with freedom in the design: a) workflow to generate the channels inside the chip: i) printing, ii) filling the channels with media and sealing the reservoirs, iii) casting the pre‐solution hydrogel inside the central chamber followed by crosslinking, iv) dissolution of PcycloPrOx filaments, v) obtainment of perfusable channels. By using this workflow, three examples of different design were produced: filaments printed inside the chips b) and their top‐view c) and 3D d) visualization of the obtained channels, after dissolving PcycloPrOx, through confocal fluorescence imaging by injecting FITC‐dextran solution (scale bar: 2 mm).

Once filaments were printed, an easy and straightforward process was tuned to generate perfusable channels (Figure 2a). First, the channels were filled with media/PBS, needed to dissolve PcycloPrOx. After that, the reservoirs were sealed with lids (ii) to prevent that liquid‐like state hydrogel, when cast inside the chamber embedding the sacrificial fiber template (iii), can flow inside the channels pushing out media. Next, the hydrogel was cured to obtain its gelation. The reservoirs were opened and filled with media (iv), and the chip was left overnight to completely dissolve PcycloPrOx filaments, obtaining remnant‐free channels ready to be perfused (v).

Different network shapes were printed inside the chamber thanks to the conceived design of the OoC and to the printing technique of FFP, opening the possibility to personalize the vascular network inside the chip based on the necessity. Three examples of printed network design are shown in Figure 2b just after the printing: a zig‐zag filament, a two‐branch structure, and two compartments geometry. In Figure 2c,d, the following geometries are shown after dissolving the PcycloPrOx filaments and the channels were visualised through confocal microscope after perfusing FICT‐dextran solution inside them.

### 2.4 Endothelialization of Microchannels

Primary human umbilical vein endothelial cells (HUVECs) were used for the biological characterization of the generated perfusion system. After dissolution and removing the sacrificial scaffold embedded in the hydrogel matrix, the resulting channels were filled with a suspension of GFP‐HUVECs by direct injection at the inlets, and samples were left at static conditions to support cell attachment to the lumen for 24 h.

**FIGURE 3:**
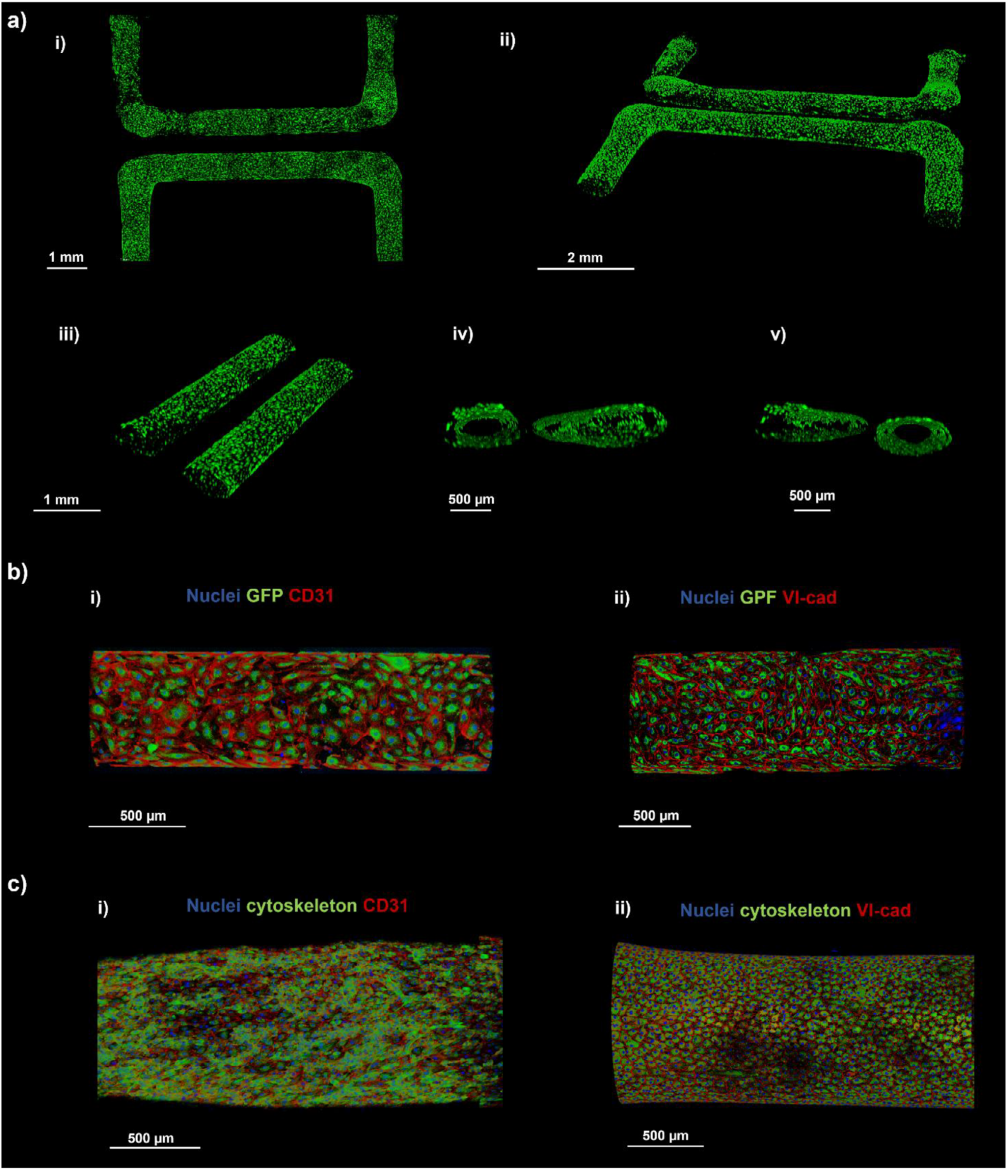
Channel endothelisation with GFP‐HUVECs. a) Endothelial cell distribution after three days of dynamic culture through the whole OoC (i and ii), zoom in the central channels (iii), and focus on the cross‐view of the channels (iv and v). In green the GFP‐HUVECs; b) Immunostaining of endothelial cells under bidirectional perfusion: i) DAPI (blue), GFP (green), CD31 (red); ii) DAPI (blue), GFP (green), VI cadherin (red). c) Immunostaining of endothelial cells under unidirectional perfusion: i) DAPI (blue), FICT‐phalloidin marking the cytoskeleton (green), CD31 (red); ii) DAPI (blue), FICT‐phalloidin marking the cytoskeleton (green), VI cadherin (red).

Perfusion was subsequently applied, and cell proliferation was monitored using confocal microscopy. At first, we studied the effect of a bidirectional perfusion. A fully confluent endothelial monolayer on the lumen of microchannels was formed on day 3 throughout the whole chip, as visible in Figure 3ai‐iii. Additionally, Figure 3a iv and v provide evidence of a circular cross‐section of the printed microvascular structures. At day 7, immunofluorescence staining was further employed to study the level of cell–cell interconnections. As shown in Figure 3b, the expression patterns of key junctional proteins (VE‐Cadherin, CD31) confirmed the formation of a confluent endothelial monolayer lining the inner channels’ surface, thus indicating the physiological formation of tight and adherent junctions.

Finally, the use of a unidirectional flow was implemented and studied. In this case, the flow was initiated after two days of static culture to allow sufficient time to the cells to adhere to the channel walls and then sustain a shear stress of 5 dyn/cm^2^. As visible in Figure 3c, in addition to junctional protein expression, after 7 days the cells appear more aligned and oriented in the direction of the flow.

## 3 Discussion

The development of 3D in vitro models possessing perfusable channels within a matrix material is a fundamental prerequisite for the construction of a wide range of functional tissue to be applied in physiological/ pathological studies, drug evaluation and regenerative medicine [14, 15]. The use of sacrificial materials has been demonstrated to be a biofabrication strategy to realize perfusable micro‐size channels embedded inside hydrogels contained in fluidic systems [16, 17, 18]. In this context, an interesting example of sacrificial material is the thermo‐responsive polymer poly(2‐cyclopropyl‐2‐oxazoline) (PcycloPrOx). Its first application in the biofabrication field was shown in the work of Ryma et al. [19], where PcycloPrOx scaffolds were created using melt electro‐writing (MEW), a technique able to generale filaments with high resolution in the micro‐scale (between 90 and 275 µm in terms of fiber diameter). The filaments were placed in a customized bioreactor system which was then filled with a hydrogel precursor solution. However, a limitation presented in this work was the fact that the scaffolds were manually glued into the perfusion chamber due to the process of MEW where an electrical field is needed to deposit the fibers. This procedure made a possible automation of the production of a vascularized in vitro model difficult. However, this problem can be solved using a 3D‐printer allowing to deposit filaments without the need of an electrical field. Particularly, thanks to its fast solidification after extruding from the printing nozzle, the printing of PcycloPrOx allows fabrication of freestanding network of cylindrical filaments defining this technique as freeform printing (FFP) [9, 10]. FFP of PcycloPrOx was first introduced by Mair et al. [13], where complex three‐dimensional geometries, such as spiral and stacked cube structures, were printed. However, no functional applications of the material were demonstrated.

In this work, we present for the first time a concrete demonstration of PcycloPrOx in combination with FFP for the generation of perfusable, endothelialized microchannels within a microfluidic OoC device specifically designed to enable direct in situ printing.

The FFP of PcycloPrOx approach offers several key advantages for vascular fabrication. First, it allows the freedom of fabricating complex microvascular patterns without contacting the chip's bottom surface, which is essential for producing circular cross‐section channels that closely mimic the geometry of native vasculature. With a resolution down to 200 µm, this method is well‐suited for reproducing perfusable microvascular dimensions relevant to tissue‐scale models.

Second, FFP of PcycloPrOx streamlines the production workflow and facilitates future integration with automated vessel‐patterning strategies, owing to its ability to be printed directly inside the chip and its elimination of labor‐intensive post‐processing steps. As a thermo‐responsive polymer that is soluble in aqueous media below 25°C, PcycloPrOx circumvents the need to physically remove molding scaffolds, such as polycaprolactone (PCL) fibers, by pulling them out of or peeling them off the hydrogel matrix [20, 21, 22]. It also avoids the creation of protective coatings around sacrificial materials, as required, for example, in freeform printing of carbohydrate glass and isomalt, due to their rapid dissolution kinetics in hydrogels [11, 12, 23, 24].

Nevertheless, the use of PcycloPrOx also imposes constraints on the choice of hydrogels, whose crosslinking timing must occur relatively quickly. PcycloPrOx fibers lose their dimensional and freestanding stability after approximately 3 min in pure water, although ongoing hydrogel gelation prolongs this timeframe by continuously stabilizing the hydrating material [13]. Hydrogels such as alginate, agarose, GelMA, and fibrin have been shown to be compatible with this polymer. However, FFP of PcyclePrOx cannot be applied to hydrogels such as unmodified gelatin, collagen, or Matrigel because of their slow gelation kinetics and insufficient mechanical stability that do not allow the formation of well‐defined channels [13, 25].

In this work, we used GelMA as proof‐on‐concept of the general approach focused on the freeform printing of PcycloPrOx for the generation of micro‐vessel in OoC. Following GelMa casting and crosslinking around the sacrificial PcycloPrOx filaments, the networks were dissolved, leaving behind perfusable microchannels. Three different examples of design were developed to show the freedom given by this technique in tuning the pattern more suitable to the user.

After selecting a two‐compartments design for the cellular validation, these channels were subsequently seeded with GFP‐labelled HUVECs and cultured under continuous perfusion for seven days. Particularly, two different types of flow were applied – i.e., a bidirectional flow generated by a rocking platform and a unidirectional flow controlled by a pump – demonstrating the versatility of our fluidic system in providing different flow modalities.

In both conditions, at day three, a complete monolayer of endothelial cells was formed along the channel walls, indicating efficient and rapid channel endothelialization. At day seven, immunostaining revealed expression of CD31 and VE‐cadherin. CD31 is a canonical marker of endothelial identity, while VE‐cadherin is a key adhesion molecule that regulates vascular permeability and intercellular junction stability. Their co‐expression in our engineered vessels indicates a correct endothelial specification and the establishment of stable, functional cell–cell junctions, which are critical for maintaining vascular integrity under flow [26, 27]. Taken together, these biological results show that our approach enables the creation of channels that are cytocompatible and able to sustain different types of flow, without leaving non‐dissolvable remnants behind.

Finally, the unidirectional flow induced a better cell alignment along the channel axis and promoted a more elongated morphology closely resembling the physiological conditions present in human vessels, being consistent with previous studies [5, 28]. This highlights that fluidic systems capable of sustaining unidirectional flow in vitro represent the most promising approach for developing models that more accurately mimic physiological human conditions and, the future steps of fluidic systems should move in this direction.

## 4 Conclusions

In summary, this study shows how FFP of PcycloPrOx enables the direct, automated fabrication of perfusable and endothelialized microvascular networks within OoC devices. This strategy bridges a major gap in microvascular engineering by providing a scalable and versatile method to reproduce physiologically relevant vascular architectures while maintaining compatibility with real‐time imaging and perfusion culture. Beyond this proof‐of‐concept, this platform has the potential to accelerate the development of vascularized OoC models for drug testing and disease modelling.

## 5 Experimental Section/Methods

### 5.1 Materials

The synthesis and polymerization of 2‐cyclopropyl‐oxazoline to PcycloPrOx with a degree of polymerization of 100 was executed as described in literature [29]. Analytical data can be found in our prior work.

Gelatin methacryloyl (GelMA) was synthesized according to the following work [30]. The precursor solution for GelMA hydrogel was created by dissolving the freeze‐dried GelMA in Phosphate‐Buffered Saline (PBS) to a final concentration of 5 wt% with 0.05 wt% Lithium‐phenyl‐2,4,6‐trimethylbenzoylphosphinat (LAP).

### 5.2 Microdevice Design and Fabrication

The chips were designed with Inventor (Autodesk) and 3D‐printed via Digital Light Projector (DLP) printer (Form 4B, Formlabs) by using a biocompatible resin (BioMed Clear Resin, Formlabs). The Computer‐Aided Design (CAD) model was exported in. stl extension and sliced with the software (Formlabs) following the Default printing parameters for BioMed Clear V1. The washing and post‐crosslinking of the printed chips were performed according to the default parameters of the resin.

### 5.3 Freeform Printing of Fiber Template

Different vascular network shapes were conceived, and the corresponding Gcode was written through Repetier‐Host software. A piston‐based 3D printer (3DBS, Brazil) was used to print PcycloPrOx with the following parameters: extrusion parameter (E): 0.08; movement speed (F): 15 mm min^−1^, temperature of the printing head 210°C. A nozzle with a diameter equal to 0.3 mm was used.

After pre‐heating for 30 min, the molten material was extruded by the piston and deposited following the designed printing path defined in the G‐code. The freestanding filaments were printed directly inside the chips.

### 5.4 Green Fluorescent Protein Human Umbilical Vein Endothelial Cells (GFP‐HUVECs) Culture

GFP‐HUVECs (Innoprot) were obtained through transient transfection with tGFP expression vector expressing the green fluorescent protein gene sequences as free cytoplasmatic protein. GFP‐HUVECs were cultured in Endothelial Cell Growth Medium 2 (EGM‐2) (Lonza) supplemented with 1% (v/v) Endothelial cell growth factor kit (Lonza), 1% (v/v) Penicillin/Streptomycin, and 10% (v/v) of Fetal bovine serum (FCS). HUVECs were discarded after 15 passages to ensure the representation of key endothelial characteristics.

### 5.5 Microchannel Formation

Organ‐on‐Chips with sacrificial fibers were sterilized under the UV light of a sterile bench (HERAsafe KS, ThermoScientific) for 60 min. After pipetting 300 uL of GelMA inside the chamber, the hydrogel was immediately crosslinked via UV‐treatment (405 nm) for 60 s.

100 uL of Endothelial Cell Growth Medium EGM‐2 media were added to the media reservoirs, and the chips were left at room temperature for 2 h to dissolve PcycloPrOx filaments. Then, the media was aspirated from the reservoirs, and cold PBS was added. After 5 min, PBS was aspirated, and new cold PBS was added. This washing step was repeated until all PcycloPrOx was dissolved, and the channels became perfusable.

Finally, 150 uL of complete endothelial growth media was added into each reservoir, and the chips were placed onto a rocker platform (MIMETAS BV Leiden, Netherlands) overnight to allow protein adsorption and complete removal of PcycloPrOx. The rocker was set for continuous perfusion at a 6° inclination and a 10‐min cycle timer.

### 5.6 Cell Seeding and Perfusion

GFP‐HUVECs suspension was prepared with a density equal to 4 × 10^6^ cells/mL inside culture media, and 100 µL of cell suspension was seeded into one of the channel inlets. After cell seeding, the chip was tilted at a 45° allowing media to flow through, and then it was left under static conditions for 30 min in a humidified CO_2_ incubator at 37°C. After 30 min, other 100 µL of cell suspension was injected into the same inlet. Then, the chip was turned upside down and incubated for further 30 min to enhance the homogeneity of the cell attachment.

The chips, cultivated under bidirectional perfusion, were placed on a rocker platform (MIMETAS BV Leiden, Netherlands) after 24 h. The rocker was set for continuous perfusion at a 6° inclination and a 10‐min cycle timer.

The chips, cultivated under unidirectional perfusion, were connected to the ibidi Pump System (ibidi GmbH) after 48 h. The ibidi pump system was set to generate a shear stress of around 5 dyn/cm^2^.

### 5.7 Immunostaining of the Endothelial Cells

After 7 days of culture, VE‐cadherin and CD31 antibody immunostaining was performed on the hydrogel constructs to investigate the inter‐cellular connection of the endothelial cells. Samples were washed three times with PBS and fixed in 4% paraformaldehyde for 10 min. The fixed constructs were washed with PBS three times and permeabilized in 0.1% Triton X‐100 for 10 min. Upon permeabilization, the samples were blocked in PBS containing 2% bovine serum albumin (BSA) for 1 h at room temperature. Anti‐CD31 (Dako,1:30) and anti‐ VE‐cadherin primary antibodies (Cell Signaling technology, 1:50) were incubated in 0.1% PBS‐BSA overnight at 4°C. Then, donkey anti‐mouse Alexa Fluor 555 (A‐315070, ThermoFisher Scientific, 1:500), goat anti‐rabbit Alexa Fluor 555 (#4413, Cell Signaling, 1:500) secondary antibodies, DAPI (300 nM) and FICT‐phalloidin (200 nM) were incubated in 0.1% BSA for 2 h in the dark at room temperature.

Constructs were washed three times with PBS and stored in PBS until imaging using a Leica DMi8 confocal microscope equipped with a Leica TCS SP8 laser.

## Conflicts of Interest

The authors declare no conflicts of interest.

## Data Availability

The data that support the findings of this study are available in the supplementary material of this article.
